# Expression of Neuron-Specific Enolase in Multiple Myeloma and Implications for Clinical Diagnosis and Treatment

**DOI:** 10.1371/journal.pone.0094304

**Published:** 2014-05-05

**Authors:** Haiping Yang, Ruihua Mi, Qian Wang, Xudong Wei, Qingsong Yin, Lin Chen, Xinghu Zhu, Yongping Song

**Affiliations:** 1 Department of Hematology, Tumor Hospital of Zhengzhou University, Zhengzhou City, China; 2 Department of Hematology, First Affiliated Hospital of Henan University of Science and Technology, Zhengzhou City, China; Federico II University, Naples, Italy

## Abstract

**Objective:**

To determine the expression of neuron-specific enolase (NSE) in patients with multiple myeloma (MM) and to evaluate its clinical value as a tumor marker and, an indicator of disease progression and treatment efficacy.

**Methods:**

Using electrochemiluminescence immunoassay (ECLIA), we measured the serum levels of NSE in 47 healthy subjects (control group), 25 patients with small cell lung cancer (lung cancer group), and 52 patients with MM (MM group). For the MM group, serum NSE levels were measured and other disease indicators and related symptoms were monitored before and after chemotherapy. The relationship between NSE expression and other MM-related factors was analyzed. In addition, immunohistochemical staining was performed on bone marrow biopsy specimens from patients with MM.

**Results:**

In the control group, serum NSE levels were within the normal range as previously reported, while the lung cancer group and the untreated MM group exhibited NSE levels that were significantly higher relative to the control group (P<0.05). The difference in NSE expression between the lung cancer group and untreated MM group was statistically significant (P<0.05). NSE levels were significantly decreased in MM patients after chemotherapy and were positively correlated with an MM disease index [beta-2 microglobulin (β_2_-MG)]. Changes in NSE were not related to the response rate to chemotherapy but rather were correlated with progression-free survival.

**Conclusions:**

Patients with MM may have increased serum NSE levels, and changes in NSE may provide insight into treatment efficacy of chemotherapy and disease progression. Perhaps NSE expression is a viable biomarker for MM and can be a useful reference for the design and adjustment of clinical MM treatment programs.

## Introduction

Multiple myeloma (MM) is a malignant plasma cell disease typified by clonal plasma cells in the bone marrow (plasma cell neoplasms) and is associated with end-organ damage, including bone damage, and the presence of monoclonal protein (M protein) in the serum or urine [1]–[4]. Treatment efficacy and recurrence can be monitored by measuring the proportion of plasma cells in bone marrow by puncture or biopsy, M protein levels in serum and urine, immune electrophoresis, and the range, number and progression of osteolytic lesions [5]. Also, the levels of blood beta-2 microglobulin (β_2_-MG), albumin, and urine light chain are used to determine therapeutic efficacy and disease progression [6]. The natural disease course of MM ranges widely from a few months to more than 20 years, and the response to treatment is variable. Recently, functional imaging tools, such as F-18 fluorodeoxyglucose (FDG) positron emission tomography (PET), have been considered for the assessment of responses [7]. However, application of this technique is quite limited due to the high cost. Therefore, the key to treatment success is to offer patients with an accurate prognosis and to adopt the appropriate treatment strategy after diagnosis.

It is becoming increasingly apparent that the identification of tumor markers is valuable in the diagnosis and treatment of various diseases [1]. Indeed, some markers have become important inference indices for cancer patients. For instance, in lung cancer, tumor markers can aid in the diagnosis of pathological type, stage, metastasis, recurrence, and prognosis. Neuron-specific enolase (NSE) is one of these markers and its application in clinical practice has been gradually increasing in recent years with significant diagnostic value [2]–[4].

Enolase is an enzyme that catalyzes the decomposition of glycerol in the glycolytic pathway and consists of three subunits (α, β, γ) and five isozymes (αα, ββ γγ, αγ, βγ) [3]. The isozymes containing a γ subunit are found in neuronal and endocrine tissue, and thus are known as the neuron-specific enolases (NSE). NSE has been implicated in tumorigenesis with neuroendocrine origin. Japanese scholars Jimbo *et al.* [1] and Nakajima *et al.* [2] and British scholars Sharma *et al.* [3] each reported a case where a patient with MM exhibited increased levels of NSE. In China, there are very few reports evaluating NSE levels in MM patients. Zhang *et al.* [4] reported that patients with MM who had increased NSE levels had a poorer prognosis than those patients with normal NSE levels. Patients with elevated NSE levels exhibited shorter overall survival and decreased progression-free survival. Moreover, although there was no correlation between NSE expression level and age, gender, M protein type, hemoglobin, or serum creatinine, there was a significant correlation between NSE expression and the abundance of myeloma plasma cells and blood β_2_-MG expression level [4]. COX analysis suggested that the levels of NSE and β_2_-MG are two independent prognostic factors that affect the survival of MM. Gao *et al.* [8] reported that NSE expression was increased in the U266 myeloma cell line and in 67% of MM patients. In addition, NSE expression trended upward as disease severity progressed and the degree of bone destruction increased. In this study, we examined the level of NSE in 52 MM patients before and after chemotherapy. In addition, we monitored the disease condition and efficacy of therapeutic intervention. Taken together, we sought to determine the relationship between NSE and MM, and to evaluate the viability of NSE as a biomarker for the diagnosis, treatment evaluation, and prognosis of MM.

## Patients and Methods

### 1 Subjects

#### 1.1 Control group

Forty-seven healthy were included in the control group and underwent physical examination. The group consisted of 29 males and 18 females with a median age of 37 (28–59) years old. ECLIA was used to detect tumor biomarkers in the following systems: respiratory, digestive, genitourinary, endocrine systems, etc. The physical examination included imaging, blood test, biochemistry analysis, infectious disease, immunization, electrocardiogram, and other tests. Those individuals without abnormalities in these tests were enrolled in the healthy control group.

#### 1.2 Small cell lung cancer group

Twenty-five patients with small cell lung cancer were included in this group. These patients were hospitalized in the Department of Medical Oncology of our hospital between March 2009 and August 2010. They had clear pathological diagnosis and were composed of 21 males and four females with a median age of 53 (36–80) years old.

#### 1.3 MM group

All 52 patients in this group were hospitalized with MM in either our hospital or the First Affiliated Hospital of Henan University of Science and Technology between May 2010 and April 2013, including 26 males and 26 females with a median age of 53 (47–62) years old. Hospitalization examinations were performed to confirm the diagnosis of MM, according to the diagnostic criteria defined in references [5], [6]. These included bone marrow biopsy, ECT whole body bone scan (or CT, PET-CT, or other imaging methods), blood count, blood chemistry, blood tumor marker testing, serum protein electrophoresis, and immunofixation electrophoresis.

#### 1.4 MM treatment programs

All MM patients were treated with either TD (thalidomide and dexamethasone) or VD (Velcade plus dexamethasone)-based programs for three courses of chemotherapy, combined with or without mitoxantrone, THP topiramate Star, cyclophosphamide, and etoposide. In patients with elevated NSE levels, 28 adopted the TD-based program (NSE+/T) and six adopted the VD-based program (NSE+/V). In patients with normal NSE levels, 14 adopted the TD program (NSE-/T) and four adopted the VD program (NSE-/V). The difference in choosing either program between the NSE+ and NSE- patient groups was not statistically significant (P = 0.723).

### 2 Research Methods

#### Ethics statement

Informed consent was obtained from all the patients in writing prior to enrollment in the study. This study was performed in strict accordance with the ethical guidelines of the Declaration of Helsinki, and the protocol was approved by the institutional Ethics Committee of Henan Cancer Hospital.

#### 2.1 Equipment

Roche Elecsys 2010 (Basel, Switzerland) was used for electrochemiluminescence detection. KDC-2046 low-speed refrigerated centrifuge (Henan, China) was purchased from Anhui Zhongjia Co., Ltd.

#### 2.2 Reagents

Reagents specific for ECLIA detection on Roche Elecsys 2010 included NSE detection kit, cleaning fluid (ISE Cleaning Solution), system reagent (Procell), standard solution, and quality control reagent (PreciControl Tumor Marer).

#### 2.3 NSE detection method

For ECLIA detection of NSE, four ml of fasting blood was taken from patients in the morning prior to eating and drinking. After coagulation, blood serum was separated at 3000 rpm, and NSE concentration was measured within two hours after separation in strict accordance with the user manual guidelines of the Roche Elecsys 2010 and the NSE electrochemical luminescence detection kit. NSE levels were read automatically on Elecsys 2010. As for detection value criteria, the normal detection range was set from zero to 15 ng/ml, and any values beyond the normal range were considered positive.

In addition, NSE levels were examined by immunohistochemistry in bone marrow biopsy specimens from patients with previously untreated MM. All tissues were fixed in 10% neutral formalin and paraffin-embedded after routine dehydration. Five to six serial sections were prepared with a thickness of four µm. Sections were dewaxed and treated with fresh 3% H_2_O_2_ to block endogenous peroxidase. After rinsing three times with PBS for three min, citric acid antigen retrieval was performed under high pressure. The slices were blocked with normal goat serum for 30 min at room temperature to eliminate nonspecific staining, followed by incubation with primary antibody solution (Abcam, Cambridge, UK) at 4°C overnight. After recovery for 40 min at room temperature, ready-to-use secondary antibody solution (rabbit anti-mouse secondary antibody, Zhongshan Golden Bridge, Beijing, China) was added dropwise to the slices and incubated at room temperature for 40 min followed by three PBS washes for 3 min. DAB reagent was added dropwise to the slices afterwards and developed at room temperature. Slices were observed under a microscope for three to five min to determine the optimal developing time, after which slices were rinsed with tap water, stained with hematoxylin for 90 seconds, differentiated with the hydrochloric acid solution, and treated with saturated aqueous lithium carbonate for blue nuclear staining. Slices were mounted with neutral gum after routine dehydration and observed under a microscope. A positive result was determined by evaluating both staining intensity and positive rates. If the positive rate was less than 10% with weak staining, it was labeled as negative; if the positive rate was greater or equal to 10% with strong brownish-yellow granules, it was labeled as positive.

We also used reverse transcriptase polymerase chain reaction (RT-PCR) to detect NSE transcript levels in the bone marrow of patients. Two ml bone marrow samples were extracted by bone marrow biopsy from previously untreated MM patients and control healthy subjects. Mononuclear cells were enriched by density gradient centrifugation. Trizol extraction of total RNA was performed followed by PCR (Takara DRR002B). The upstream primer sequence for NSE: 5′-GACTGAGGACACATTCATTGCTGAC-3′; downstream primer sequence: 5′-CAGCACACTGGGATTACGGAAG-3′. Eight µl reaction product together with 2 µl loading buffer was resolved on a 2% agarose ethidium bromide (EB)containing gel by electrophoresis. Results were documented under a UV transmission reflectometer.

### 3 Monitoring of patient condition indices

Prior to each course of chemotherapy, weekly routine preoperative examinations were performed on each patient. These included blood count, liver function, renal function, β_2_-MG, serum NSE, serum protein electrophoresis, immunofixation electrophoresis, serum immunoglobulin (IgG, IgA and IgM) quantification, light chain (κ,λ) quantification, and bone marrow cell morphology. Meanwhile MM-associated symptoms, such as bone destruction, infection, high viscosity syndrome, anemia, hypercalcemia, and renal damage, were monitored and recorded.

### 4 Statistical analysis

SPSS16.0 software (Armonk, NY, USA) was used for statistical analysis. Because NSE level data in the control group, lung cancer group, and MM group exhibited a skewed distribution, percentiles were chosen for data presentation. Comparisons between the lung cancer group and control group, the MM group and control group, and the lung cancer group and the MM group were analyzed by rank-sum test. The correlation between NSE level in MM patients and the amount of the prognostic indicator β_2_-MG was analyzed by Spearman's rank test.

## Results

### 1

Among the 52 MM patients evaluated, 34 exhibited increased serum NSE levels, accounting for 65.4% of all patients with MM. Spearman's statistical analysis showed r = 0.692, p<0.05 (Table 1), indicating that NSE serum levels in MM patients were positively correlated with IHC results.

**Table 1:** Correlation analysis of serum NSE levels and IHC results.

Patient No.	1	2	3	4	5	6	7	8	9	10	11	12	13
NSE (ng/ml)	14.34	11.89	14.34	23.43	25.64	33.43	80.34	32.54	13.46	38.54	25.43	50.32	28.65
IHC result	-	-	-	+	+	+	+	+	+	+	+	+	+
Patient No.	14	15	16	17	18	19	20	21	22	23	24	25	26
NSE (ng/ml)	22.43	12.43	14.75	24.86	24.3	38.3	9.643	14.2	12.64	40.4	26.79	49.17	29.6
IHC result	-	-	+	+	+	+	-	+	-	+	-	+	+
Patient No.	27	28	29	30	31	32	33	34	35	36	37	38	39
NSE (ng/ml)	23.77	21.48	13.64	28.96	12.54	11.65	13.87	13.26	16.88	7.23	22.76	20.21	27.82
IHC result	+	+	-	+	-	-	+	-	-	-	+	+	+
Patient No.	40	41	42	43	44	45	46	47	48	49	50	51	52
NSE (ng/ml)	25.12	30.54	12.53	33.05	21.16	33.98	79.27	13.68	20.04	8.65	21.12	30.78	25.38
IHC result	+	+	-	+	+	+	+	-	+	-	+	+	+

Note: +, IHC result positive; -, IHC result negative.

Spearman's statistical analysis shows rr = 0.692, p<0.05, indicating that NSE serum levels in MM patients were positively correlated with the IHC results.

### 2 Comparison of serum NSE levels between the control and small cell lung cancer groups (Table 2)

As shown in Table 2, the serum NSE level in the control group all fell within the normal range, whereas the level in lung cancer group were significantly higher at the level of third percentile (P<0.05).

**Table 2:** Comparison of serum NSE levels between small cell lung cancer group and control group.

			Percentile					
Indicator(ng/ml)	Group	Cases(n)	25th	50th	75th	Range	Z	P
NSE	Control	47	8.11	9.33	10.8	5.73–14.40	6.937	P<0.05
	Lung cancer	25	30.86	44.6	95.85	13.91–370.0		

### 3 Comparison of serum NSE levels between the control and MM groups (Table 3)

As shown in Table 3, NSE serum levels in 18 cases in the MM group were negative. However, at the level of third percentile, NSE levels of the MM group were significantly higher than those of the control group (P<0.05).

**Table 3:** Comparison of serum NSE levels between MM group and control group.

			Percentile					
Indicator(ng/ml)	Group	Cases(n)	25th	50th	75th	Range	Z	P
NSE	Control	47	8.11	9.33	10.8	5.73–14.40	5.356	P<0.05
	Lung cancer	52	13.72	23.10	30.31	7.23–880.34		

### 4 Comparison of serum NSE levels between the small cell lung cancer and MM groups (Table 4)

As shown in Table 4, at the level of third percentile, NSE levels in the small cell lung cancer group were significantly higher than those of the MM group (P<0.05).

**Table 4:** Comparison of serum NSE levels between small cell lung cancer group and MM group.

			Percentile					
Indicator(ng/ml)	Group	Cases(n)	25th	50th	75th	Range	Z	P
NSE	Lung cancer	25	30.86	44.6	95.85	13.91–370.0	2.739	P<0.05
	Lung cancer	52	13.72	23.10	30.31	7.23–880.34		

### 5

**Figure 1:**
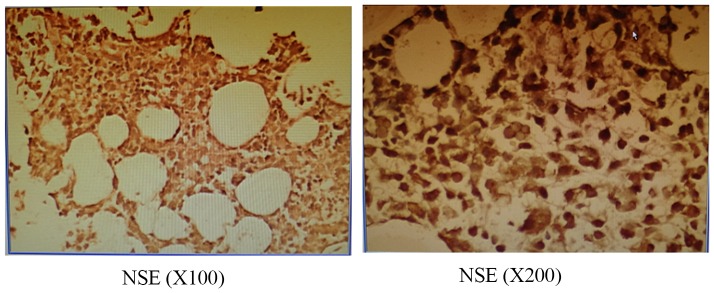
NSE immunohistochemical results of patients with previously untreated MM.

Immunohistochemistry for NSE in patients with previously untreated MM (Figure 1) reveals clear brownish-yellow granules in cytoplasm with a positive rate of 45%.

### 6

**Figure 2:**
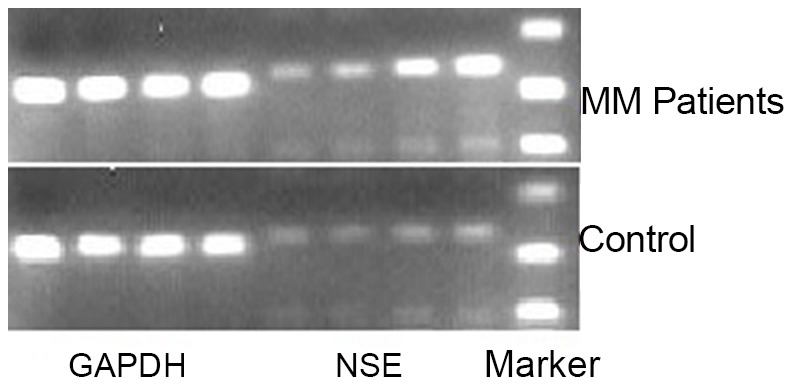
RT-PCR product electrophoresis for GAPDH (control and NSE) in previously untreated MM patients and controls.

RT-PCR was used to determine if RNA transcript levels of NSE were elevated in the bone marrow of untreated patients diagnosed with MM. Relative to healthy controls, MM patients exhibited elevated levels of NSE. GAPDH was used as an internal control, and its levels were unchanged between the two groups (Figure 2).

### 7 Correlation between NSE level in patients with previously untreated MM and the amount of the prognostic indicator β_2_-MG (Figure 3)

According to Spearman's rank test, r = 0.749, P<0.01, there is a significant positive correlation between β_2_-MG and NSE levels in MM patients.

**Figure 3:**
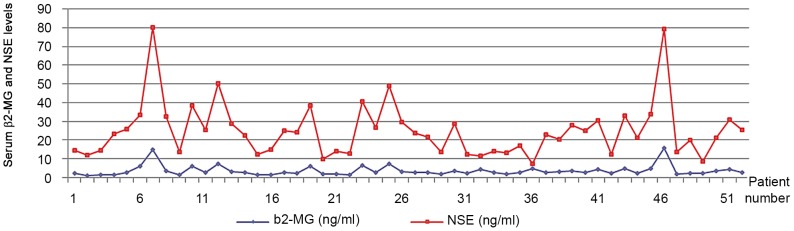
Correlation between NSE level in patients with previously untreated MM and the amount of the prognostic indicator β2-MG.

### 8 Correlation between NSE level and the amount of prognostic indicator β_2_-MG in MM patients after receiving three courses of chemotherapy (Figure 4)

According to Spearman's rank test, r = 0.618, P<0.01, there is even after chemotherapy a strong positive correlation between β_2_-MG and NSE levels in MM patients.

**Figure 4:**
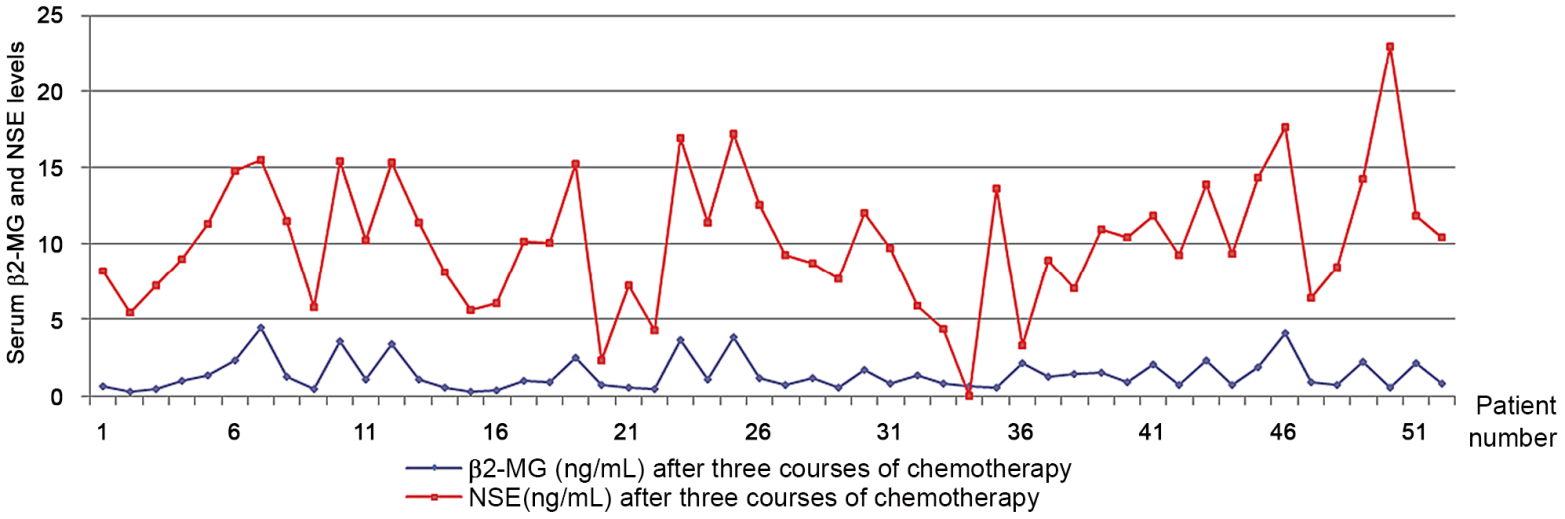
Correlation between NSE level and the amount of prognostic indicator β2-MG in MM patients after receiving three courses of chemotherapy.

### 9 Correlation between NSE level in MM patients and treatment response

After three courses of chemotherapy, the overall response rate (ORR) of the group with elevated NSE levels was 15/28 (53.6%), and three out of 28 patients (10.7%) achieved very good partial response (VGPR). In the group with normal NSE levels, the ORR was 10/14 (71.4%) and two patients (14.3%) achieved VGPR. In the group of patients with elevated NSE levels who adopted the TD-based program (NSE+/T group), the treatment was effective in 15 patients and the ORR was 53.6%. In the group of patients with elevated NSE levels who adopted the VD-based program (NSE+/V group), the treatment was effective in four patients and the ORR was 66.7%. In the group of patients with normal NSE levels who adopted the TD-based program (NSE-/T group), the treatment was effective in 10 patients and the ORR was 71.4%. In the group of patients with normal NSE levels who adopted the VD-based program (NSE-/V group), all four patients showed effective response. The difference in treatment efficiency between groups with elevated serum NSE levels and the ones with normal NSE levels was not statistically significant (P>0.05).

### 10 Correlation between NSE level in MM patients and progression-free survival (PFS)

**Figure 5:**
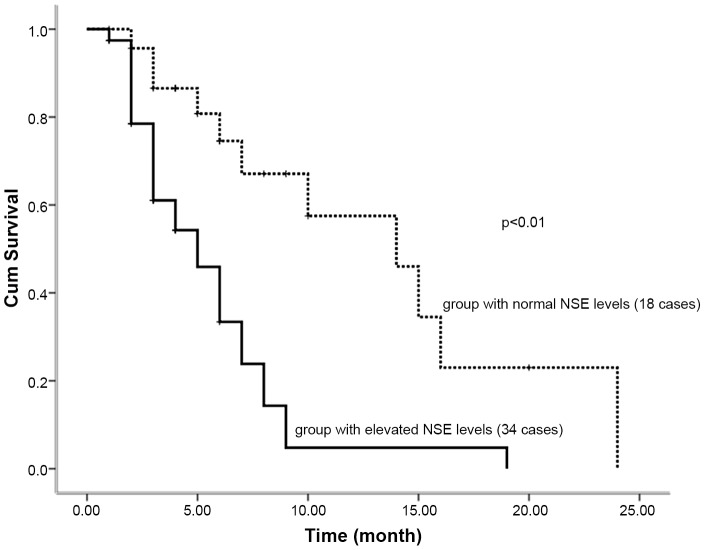
Correlation between NSE level in MM patients and progression-free survival (PFS).

The median PFS in patients with elevated and normal serum NSE levels was five months and 13 months, respectively. The difference in PFS was statistically significant (P<0.01) (Figure 5).

## Discussion

To date, there are only a few published studies regarding elevated NSE levels in MM. Jimbo *et al.* [1] reported increased serum NSE level in a 53-year-old female patient diagnosed with IgG-λ type MM with a chest-wall plasmacytoma. Immunostaining of her bone marrow smears and left chest-wall tumor biopsy specimens revealed diffused cytoplasmic NSE staining in the abnormal plasma cells, confirming that myeloma cells can produce NSE. After several cycles of chemotherapy, along with the disappearance of chest-wall plasmacytoma and plasma cells in the bone marrow, her serum NSE level returned to normal. Coincidentally, another Japanese group [2] reported a case of a 68-year-old patient with IgD-λ type MM exhibiting significantly elevated levels of serum NSE. Immunohistochemical staining confirmed NSE expression in myeloma cells. NSE level in this patient was reduced to normal after two cycles of combined interferon-α and vincristine, melphalan, cyclophosphamide, and prednisone (VMCP) chemotherapy. British scholars Sharma *et al.* [3] reported a case of a 75-year-old male patient who was initially hospitalized with sacral pain was diagnosed with prostate cancer. Following chemotherapy, the patient presented with multiple sites of bone pain, hypercalcemia, positive urine for B-J proteins, and elevated serum NSE. Bone marrow biopsy showed atypical plasma cells comprising 20–30% of the nucleated cells. In addition, immunohistochemical staining showed positive staining for CD138, κ light chain, and NSE. The patient was diagnosed with IgG-κ type MM and was treated with cyclophosphamide, thalidomide, and dexamethasone. Moreover, Japanese scholars reported detection of NSE expression in MM cell lines and primary cells by immunohistochemistry and PCR, further confirming the association of NSE expression with MM [9].

In the present study, 34 of the 52 MM patients examined showed elevated NSE levels in the initial detection of NSE. Following chemotherapy, NSE levels exhibited a downward trend. This was particularly true in patients treated with Velcade, a finding consistent with the downward trend of another MM monitoring indicator blood β_2_-MG concentration. There was a significant positive correlation between NSE and β_2_-MG levels. Although no significant correlation was detected, we observed that elevated NSE levels were often present in patients with severe bone pain symptoms or when the symptoms worsened. In contrast, NSE levels were not significantly related to other MM symptoms, such as anemia, hyperviscosity, and hypercalcemia. Consistent with previous reports, it is important to note that the PFS of patients with elevated NSE levels was significantly shorter than patients with normal levels of NSE. However, the overall survival data was not included for analysis since in all cases the observation time was less than three years, and the tumor burden in patients with disease progression had decreased to some extent after induction of remission therapy. These patients continue to be followed clinically, and the total sample size will continue to expand in order to study the correlation between NSE level and five year overall survival and the impact of different treatment programs on NSE level.

We also observed with the conduct of chemotherapy that MM indices such as proportion of plasma cells and M protein level declined. In parallel, individual NSE levels in each patient also decreased, suggesting that it can be used as an indicator for condition monitoring. The reason for the decline in NSE level with chemotherapy could be that during the process of tumor cell growth, the cell cycle is accelerated and glycolysis is strengthened. NSE is an acidic protease that is involved in glycolysis to catalyze the conversion of β-glycerophosphate into dihydroxy acetone phosphate. Therefore, the upregulation of intracellular NSE in tumor cells leads to increased release of NSE into the blood and results in increased level of serum NSE. Mature plasma cells are the dominant tumor cell type in the majority of myeloma, and the proportion of cells remaining in the cell division cycle is very small [10]. Plasma cell labeling index (PCLI) is a representative of plasma cell DNA synthesis and reflects the progression state of myeloma, and it is an important indicator of prognosis of patients with MM [11]. We speculate that, similar to PCLI, NSE does not primarily reflect the overall myeloma cell load but dynamically reflects the proliferation of myeloma cells. Therefore, NSE levels may become a new prognostic indicator. Importantly, since detection of NSE is simple and relatively inexpensive, NSE may be a more valuable clinical application than PCLI and can be incorporated into the routine examination of neoplastic diseases.

Consistent with previous studies, we found that the level of NSE was significantly higher in patients with untreated small cell lung cancer. It is widely agreed that the NSE level is a reliable indicator for the differential diagnosis of small cell lung cancer and a useful measure for the monitoring of the therapeutic efficacy of radiotherapy and chemotherapy [12]–[15]. For diagnosis of neuroblastoma, sensitivity of NSE measurements can be up to 90% and have also been used to monitor treatment efficacy and relapse [16]. Increased NSE expression has also been linked to a small number of tumor cell lines, including non-small cell lung cancers, medullary thyroid carcinoma, and pheochromocytoma [17]. Several studies have found that NSE can be used as a sensitive and specific indicator for brain damage, and increases in NSE reflect the size and severity of brain damage [18]–[19]. An increase in NSE has been found in cerebral ischemia and neuronal injury due to a variety of reasons, such as neonatal asphyxia, pediatric febrile seizures, brain infectious diseases, chronic obstructive pulmonary disease, cerebral infarction, cerebral hemorrhage, systemic lupus erythematosus, Wilson's degeneration, and depression. Bai *et al.* reported that the NSE levels in patients with lymphoma were significantly increased [20]. In addition, increased NSE was seen in patients with extramedullary hemolysis, such as autoimmune hemolytic anemia and paroxysmal nocturnal hemoglobinuria, and can be used as a diagnostic indicator to distinguish *in situ* and extramedullary hemolysis [21]. However, even though there was a multitude of research regarding NSE levels in numerous types of cancer and other disease, there was little data available in the Chinese literature regarding NSE levels in MM. One study by Zhang *et al.* [5] reported that MM patients with increased NSE levels had shorter overall survival, less progression-free survival, and a poorer prognosis than those with normal NSE levels. Consistent with this report, we observed in our study that the PFS of patients with elevated NSE levels was significantly shorter than patients with normal levels of NSE.

Given our data regarding the correlation between NSE level and MM condition changes and in consideration of the above-mentioned studies abroad, we propose that serum NSE levels in patients with multiple myeloma can be increased to varying degrees. NSE levels may not be useful for MM diagnosis or therapeutic evaluation but for the prognosis. However, due to the limited number of cases in this study, confirmation of our conclusions regarding the use of NSE as a prognostic indicator in multiple myeloma will require long-term, large-scale prospective clinical observation.
